# Effects of Different Blood Flow Restriction Training Modes on Body Composition and Maximal Strength of Untrained Individuals

**DOI:** 10.3390/life14121666

**Published:** 2024-12-16

**Authors:** Hualong Chang, Xudong Yang, Biao Chen, Jianli Zhang

**Affiliations:** 1 College of Physical Education and Health Sciences, Zhejiang Normal University, Jinhua 321004, China; 2 Department of Sports Science, Chungnam National University, Daejeon 34134, Republic of Korea; 3 Renji College, Wenzhou Medical University, Wenzhou 325035, China; 4 Exercise and Metabolism Research Center, College of Physical Education and Health Sciences, Zhejiang Normal University, Jinhua 321004, China

**Keywords:** blood flow restriction, maximal strength, body composition, absolute cuff pressure, incremental cuff pressure

## Abstract

Background: The objective of this study was to examine the impacts of absolute cuff pressure blood flow restriction (A-BFR) training and incremental cuff pressure blood flow restriction (I-BFR) training, under equal cuff pressures, on body composition and maximal strength among untrained adults. Additionally, we aimed to compare these effects with those observed in high-load resistance training (HL-RT). Methods: Thirty-three adults without prior professional sports or resistance training experience were recruited and randomly assigned to three groups (*n* = 11 per group) for an 8-week training program, held three times weekly. The A-BFR group trained with a 20% 1RM load and a cuff occlusion pressure set at 190 mmHg. The I-BFR group initiated training with an occlusion pressure of 160 mmHg, which incrementally increased by 20 mmHg every two weeks, with other conditions mirroring those of the A-BFR group. The HL-RT group trained with a 70% 1RM load. Results: All three groups demonstrated a statistically significant improvement in lower-body maximal strength (*p* < 0.01), with no significant differences observed among the groups (*p* > 0.05). A notable increase in left-leg muscle mass was seen across all groups (*p* < 0.05). However, total muscle mass, right-leg muscle mass, fat-free mass, BMI, bone mineral density, and bone mineral content remained relatively unchanged (*p* > 0.05), with no significant differences among the groups (*p* > 0.05). Only the HL-RT group exhibited a significant increase in left-leg thigh circumference (*p* < 0.05), while right-leg thigh circumference remained stable (*p* > 0.05), with no significant intergroup differences (*p* > 0.05). Conclusions: While A-BFR and I-BFR did not yield statistically significant differences in overall training outcomes, A-BFR demonstrated a slightly stronger potential. A-BFR and I-BFR achieved comparable gains in muscle strength and improvements in body composition to those seen with HL-RT. However, HL-RT demonstrated more significant improvements in leg circumference.

## 1. Introduction

Blood flow restriction (BFR) training, also referred to as KAATSU training, is a training technique that utilizes pressurized devices during exercise to accomplish resistance objectives. Its primary advantage is the ability to mimic the effects of high-load resistance training (HL-RT) through the combination of low-load resistance exercises and pressurization [1,2], thereby minimizing the risk of training-related injuries. The underlying principle involves restricting blood flow via pressure bands, which triggers a cascade of muscular reactions, ultimately leading to enhanced muscle strength and improved body composition [3,4]. For example, studies have shown that after 4 weeks of KAATSU training, the weight of participants in the BFR group increased significantly, with mass, hip circumference and right-thigh circumference also increasing significantly [5]. Furthermore, BFR training has been shown to bolster participants’ muscle fitness [6], encompassing maximum strength gains in upper-body exercises such as the bench press and lat pulldown, as well as in lower-body exercises like squats, leg presses, knee flexion, and extension [7,8]. Meanwhile, BFR training can also enhance aerobic capacity, prevent disuse muscle atrophy, expedite the recovery process following injuries and surgeries, and potentially have a positive impact on the treatment of musculoskeletal-related disorders [9].

While BFR training offers certain benefits, it also poses certain risks. These risks are primarily manifested in the following three areas: In terms of blood vessels and blood, excessive pressure, tightness, or duration during pressure training can compress blood vessels, leading to poor blood flow, blood stagnation, and edema. Over time, this may also affect blood flow patterns, increase platelet adhesiveness, elevate blood coagulation, and even damage blood vessel walls, ultimately triggering venous thrombosis and thrombophlebitis [10,11]. Regarding the cardiovascular and respiratory systems, prolonged pressure training with pressures exceeding the body’s tolerance can significantly increase the risk of blood clot formation in frail individuals. Once these clots form, they may obstruct blood vessels, thereby elevating the risk of conditions such as pulmonary embolism and myocardial infarction [10,11]. Furthermore, inappropriate pressure protocols may increase peripheral resistance, decrease effective blood volume, and lower blood pressure, inducing systemic responses such as tachycardia and tachypnea [10,12]. In terms of neurological sensations, improper pressure application during pressure training may irritate nerve fiber bundles, causing ischemic hypoxia in nerve tissue, which in turn can lead to neural paralysis and slowed conduction velocities. Patients may experience symptoms such as limb numbness and, in severe cases, may even suffer from syncope due to cerebral hypoperfusion [10,13].

Previous research has established that BFR training can effectively boost muscle strength and physical fitness with reduced exercise loads [2,6,14]. However, in practical applications, the setting of BFR training parameters, such as load, frequency, and rest intervals, is crucial as these directly influence the training outcomes. Among these parameters, maintaining a training load at 20–40% of 1RM (one repetition maximum), ensuring a high training volume (approximately 75 repetitions, consisting of 30 repetitions in the initial set and 15 repetitions in each of the following three sets), a training frequency of two to three times weekly, and managing cuff pressure during rest intervals [15,16], are advantageous for participants. Within the academic community, there are differing views on the application of cuff pressure. Some scholars favor the use of absolute pressure [8,17,18], while others advocate for progressive pressure [19,20]. A recent meta-analysis indicated that the method of cuff pressure application has a specific impact on training benefits [21], yet few studies have investigated whether these two pressure application methods affect training outcomes when other factors remain consistent.

Therefore, this study established an incremental pressure blood flow restriction (I-BFR) training group and an absolute pressure blood flow restriction (A-BFR) training group, ensuring that the overall cuff pressure was equivalent for the short-term intervention training. Additionally, an HL-RT group was established as a traditional control group. This study observed the changes in body composition and maximum strength before and after the intervention in the various resistance training groups; it explored BFR exercise prescriptions suitable for untrained adults and provided theoretical references and a practical foundation for BFR training in fitness programs for the general population.

## 2. Materials and Methods

### 2.1. Participants

The inclusion criteria for study participants were as follows: (1) non-athletic young adults aged 18 to 25 years with a BMI between 18.5 and 25.0; (2) individuals with no prior history of professional athletic training or lower-body resistance training; (3) healthy individuals without a history of metabolic, hormonal, orthopedic, or cardiovascular disorders.

Thirty-three eligible subjects were successfully recruited from local universities. Prior to the study’s official start, detailed explanations regarding safety measures and emergency response protocols during the testing procedures were provided to ensure that all participants fully understood and voluntarily participated in the study. Each participant was assured of the confidentiality of their personal information and was explicitly informed of their right to withdraw from the study at any time during the intervention period for personal reasons. All subjects voluntarily signed an informed consent form and completed the “Physical Activity Readiness Questionnaire” with complete understanding. The study adhered to the principles of the Declaration of Helsinki. Ethical approval for this study was granted by the Ethics Committee of Zhejiang Normal University (approval number: ZSRT2023034). In terms of experimental design, a gender-stratified random sampling approach was employed to allocate participants to three resistance training groups, with each group comprising 11 subjects. The basic demographic information of the participants is presented in Table 1.

### 2.2. Training Protocols

This study spanned 10 weeks, with participants becoming familiar with the testing and exercise protocols 3 to 7 days before the intervention began. The testing involved evaluations of body composition and one-repetition maximum (1RM) strength for knee extension, knee curl, and leg press. These assessments were conducted both before training (as a baseline) and at the study’s conclusion, with each session lasting approximately 2 to 2.5 h. Researchers closely monitored the entire testing process to guarantee data accuracy and participant safety.

**Figure 1:**
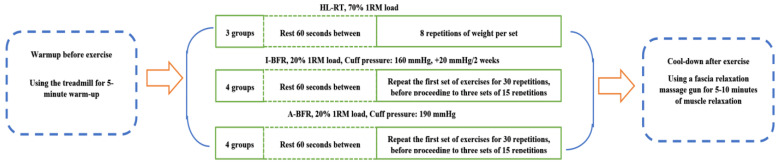
Flowchart of the Training Intervention.

The intervention period lasted for eight weeks, with training sessions held three times weekly, encompassing exercises such as leg press, knee extension, and knee flexion. Throughout the study, all participants were instructed to maintain a controlled diet to ensure the precision and reliability of the research findings. The training process is shown in Figure 1. Prior to each training session, participants engaged in a standard 5 min warm-up. The HI-RT group utilized a load of 70% 1RM, with 1 min rest periods between sets. Each exercise was performed in three sets of eight repetitions.

In this study, the A-BFR group maintained a cuff pressure of 190 mmHg throughout the training, whereas the I-BFR group started at 160 mmHg and incremented by 20 mmHg every two weeks until reaching 220 mmHg. The restriction pressure was selected in accordance with previous studies [3,19,22]. The BFR groups used a load of 20% 1RM, with 1 min rest intervals between sets. Each exercise was performed in four sets, with the first set consisting of 30 repetitions and the subsequent three sets each containing 15 repetitions. In the fourth week, all groups underwent a re-evaluation of their 1RM with the aim of establishing new training loads tailored to each participant’s progress. Participants in the BFR groups wore specialized BFR training devices (KAATSU MASTER, Kaatsu Japan Co., Ltd., Tokyo, Japan) and portable smart BFR instruments (YIDONGKANG ZNJY-01, Yijiayuan Sports Technology Development Co., Ltd., Beijing, China). The cuffs remained inflated during training, measuring 5 cm in width. In this study, pressure was applied to both limbs, with the compression site positioned at the upper one-third of the thighs. All resistance exercises were controlled by a metronome to ensure a 2 s concentric and eccentric contraction phase.

### 2.3. Muscular Strength Testing

The subject’s estimated 1RM was initially determined based on their subjective feelings, and a warm-up weight of 50% of this estimated value was utilized. Following the completion of the warm-up exercises, participants took a 1 min rest period to recover. Thereafter, the load was incrementally increased until the subject was unable to perform a full-range repetition within five attempts. During the testing, the rest interval between 1RM attempts was determined according to the subject’s recovery status and fitness level, typically ranging from 2 to 5 min. In cases where the estimated 1RM exceeded the equipment’s weight limit, it was calculated using the following formula [23]: 1RM (kg) = submaximal repetition weight/(102.78 − 2.78 × number of maximal repetitions)/100.

### 2.4. Body Composition Testing

Muscle mass, fat-free mass, and BMI were measured using a bioelectrical impedance analysis machine (TANITA BC-720, Tanita Corporation, Tokyo, Japan). These measurements were conducted in the morning after an overnight fast. To ensure accuracy, participants were instructed to empty their bladder, refrain from consuming food and water, and avoid engaging in intense physical activity prior to the test. Prior to the test, the instrument was thoroughly calibrated. Participants were required to remove their outer clothing, shoes, and any metal objects on their person. They stood on the designated spots on the body composition analyzer, extended their arms approximately 45 degrees from their bodies, looked straight ahead, maintained a normal upright posture, and remained stationary until the test was completed.

The thigh circumference of the participants was measured using a non-elastic tape measure. Based on previous research, the measurement point was chosen at the midpoint of the thigh, which is located halfway between the hip crease and the knee. During the measurement, the tape was ensured not to compress the fat layer on the skin surface. If the difference between two measurements was 0.5 cm or more, the measurements were repeated.

Bone mineral density and bone mineral content were measured using dual-energy X-ray absorptiometry (EXA-3000, Ostéo Corporation, Seoul, Republic of Korea). Before the test, the instrument was calibrated. During the test, participants were required to remove any metal objects and hard nonmetallic accessories from their bodies.

### 2.5. Data Analysis Methods

The results are presented in the format of mean ± standard deviation ($\bar{X}$ ± S). Prior to analysis, the Shapiro–Wilk normality test was conducted, confirming that all data followed a normal distribution. Additionally, one-way analysis of variance (ANOVA) was employed to assess and compare the intergroup variations in data at baseline and post-intervention. A paired sample t-test was also used to evaluate the intragroup changes within each group by comparing pre-intervention and post-intervention data.

A statistical significance level of α = 0.05 was predetermined, meaning that any differences with a *p*-value less than 0.05 would be deemed statistically significant. Furthermore, percentage changes (Δ%) were calculated as follows: Δ% = ((post-test value − pre-test value)/pre-test value) × 100. Furthermore, the study also estimated the effect size before and after the experiment through paired-samples t-tests and selected Cohen’s d as the representation method for the effect size. Based on the values of Cohen’s d, the effect sizes were classified into three categories: small effect size (0.2 ≤ Cohen’s d < 0.5), medium effect size (0.5 ≤ Cohen’s d < 0.8), and large effect size (Cohen’s d ≥ 0.8). All statistical computations and analyses were conducted using IBM SPSS Statistics version 27.0.

## 3. Results

### 3.1. Changes in Maximal Muscular Strength Post-Training Intervention

**Figure 2:**
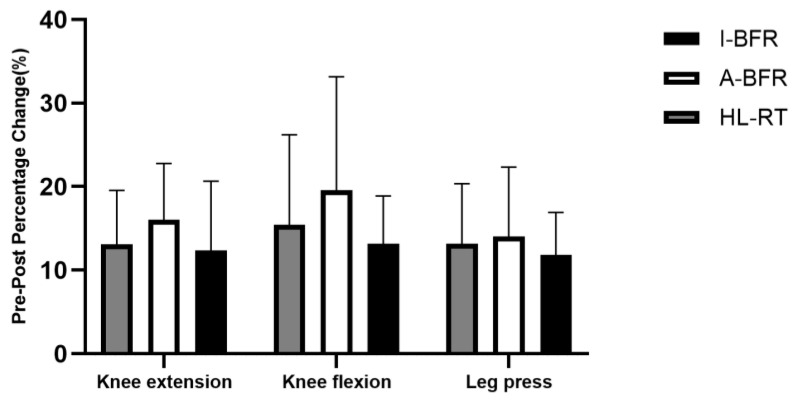
The Percentage Change (Pre-Post) following the intervention.

As demonstrated in Table 2 and Figure 2, within-group comparisons showed that the I-BFR, A-BFR, and HL-RT groups all exhibited significant increases (*p* < 0.01) in their leg press 1RM, knee flexion 1RM, and knee extension 1RM following the intervention, compared to their baseline values. When comparing between groups, no significant differences (*p* > 0.05) were found in these indices among the groups, and similarly, no significant differences (*p* > 0.05) were observed in the rates of muscular strength improvement post-training. Notably, the magnitude of maximal strength gain in the A-BFR group was slightly greater than that in the HL-RT and I-BFR groups.

### 3.2. Changes in Fat-Free Mass, BMI, and Muscle Mass

As indicated in Table 3, within-group comparisons showed that the I-BFR, A-BFR, and HL-RT groups all experienced significant increases (*p* < 0.05) in left-leg muscle mass after completing 8 weeks of resistance training intervention. However, the increases in total muscle mass, right-leg muscle mass, fat-free mass, and BMI were not statistically significant (*p* > 0.05). When comparing between groups, no significant differences (*p* > 0.05) were observed in muscle mass, fat-free mass, or BMI among the I-BFR, A-BFR, and HL-RT groups, both before and after the intervention. Additionally, there were no significant differences (*p* > 0.05) in the rates of change for these variables from pre- to post-intervention among the groups.

### 3.3. Changes in Bone Mineral Density and Bone Mineral Content

As shown in Table 4, within-group comparisons revealed that none of the experimental groups (I-BFR, A-BFR, or HL-RT) exhibited significant changes (*p* > 0.05) in bone mineral density or bone mineral content compared with their baseline values post-intervention. Inter-group comparisons revealed no significant differences (*p* > 0.05) in bone mineral density and bone mineral content were found among the groups before and after training, and no significant differences (*p* > 0.05) in the rates of change were observed post-training. Notably, participants with initially low bone mass showed an increase in bone mass after training, reaching normal ranges.

### 3.4. Changes in Thigh Circumference

As shown in Table 5, the HL-RT group experienced a statistically significant increase (*p* < 0.05) in left-leg thigh circumference after completing 8 weeks of resistance training. In contrast, the A-BFR and I-BFR groups did not demonstrate significant increases (*p* > 0.05) in left-leg thigh circumference. Similarly, none of the groups showed significant increases in right-leg thigh circumference. Before and after the experiment, there were no significant differences (*p* > 0.05) in leg thigh circumference among the groups, and no significant differences (*p* > 0.05) in the rates of change in leg thigh circumference were observed post-intervention.

## 4. Discussion

This study explored the alterations in lower-body maximal strength, body composition, and thigh circumference among previously untrained adults who underwent an 8-week program involving A-BFR, I-BFR, and HL-RT. It marks the first comparison of the training benefits between I-BFR and A-BFR. After eight weeks of consistent training, the results unveiled significant improvements in leg press, knee flexion, and knee extension maximal strength across all three groups. This outcome indicates that BFR training is on par with HL-RT in boosting lower-body maximal strength and presents a viable option for adults who, for various reasons, cannot participate in high-load training. In terms of body composition, while overall changes were not statistically significant among the groups before and after the experiment, a closer analysis revealed enhancements in specific components, such as muscle mass and bone mass. Notably, participants with initially low bone mass experienced an increase in bone mass, bringing it within the normal range. This discovery has positive implications for preventing and addressing bone insufficiency. Furthermore, all resistance training groups observed an increase in lower-limb thigh circumference, but only the HL-RT group demonstrated a statistically significant increase in left-leg thigh circumference. This result may suggest that HL-RT has a more prominent effect on enhancing lower-limb muscle morphology.

In summary, BFR training, especially A-BFR, can yield comparable gains in muscle strength and improvements in body composition to HL-RT among untrained populations. These findings hold significant implications for public health and the prevention of muscle wasting conditions, such as sarcopenia. BFR training may offer a practical alternative to traditional HL-RT for individuals who may not be able to tolerate high-load exercise.

### 4.1. Comparative Analysis of Dynamic Maximal Muscle Strength Changes

There has been ongoing academic debate about whether BFR can achieve comparable muscle strength gains to HL-RT in untrained populations. Some studies have demonstrated that BFR can match HL-RT in terms of muscle strength gains [2,24], while others have found it to be inferior [25,26]. These discrepancies are likely due to inconsistencies in exercise prescriptions. A meta-analysis found that HL-RT is typically superior to BFR in muscle strength gains, even after controlling for various factors [27]. However, another meta-analysis, which further stratified occlusion pressure prescriptions, revealed that I-BFR outperforms A-BFR in muscle strength training [21].

To validate this hypothesis, our study examined the impact of cuff pressure from different application methods on muscle strength gains, while controlling for other variables. After eight weeks, both the I-BFR and A-BFR groups showed increases in leg press 1RM, knee flexion 1RM, and knee extension 1RM—specifically, 12%, 13%, and 12% for I-BFR and 14%, 19%, and 16% for A-BFR, respectively. Although no statistically significant differences were observed among the groups, A-BFR generally exhibited higher muscle strength gains, potentially due to the high initial cuff occlusion pressure, which may have led to greater muscle activation [28,29]. Higher occlusion pressure can induce more phosphate accumulation, resulting in faster muscle fatigue and promoting faster-twitch fiber recruitment, leading to better adaptations during the initial training phase [30].

Our findings align with those of previous studies [22,25], which showed that BFR training yields similar muscle strength gains to HL-RT. However, Yasuda et al. found HL-RT to be superior, likely due to differences in training load and volume [3]. Notably, BFR training involves local compression at the proximal limb, which may cause ischemia and hypoxia [31], leading to inadequate lactate clearance [32,33]. Moderate BFR training may result in muscle damage [34], with common side effects such as subcutaneous bleeding and temporary numbness, which typically diminish upon cuff release and continued training [35]. Based on these insights, our study underscores the importance of individualizing cuff pressure selection in BFR training to ensure safety and optimize training outcomes.

### 4.2. Comparative Analysis of Changes in Body Composition

As illustrated in Table 3 and Table 4, all three groups—A-BFR, I-BFR, and HL-RT—experienced significant gains in left-leg muscle mass following the 8-week exercise intervention. However, no notable improvements were observed in other body composition metrics, including lean body mass, BMI, total muscle mass, right-leg muscle mass, bone mineral density, and bone mineral content. Traditional HL-RT promotes muscle strength and hypertrophy by increasing the number and size of myofibrils, ultimately leading to an expansion in muscle cross-sectional area [36]. The American College of Sports Medicine recommends moderate-to-high load resistance training, specifically at 60% to 80% of 1RM, for effective muscle strength gains and hypertrophy [36].

In contrast, BFR training achieves similar outcomes to high-load training but under lower loads by restricting both arterial and venous blood flow. This training method induces metabolic stress, which enhances the accumulation of metabolic byproducts like lactate within the muscles, thereby stimulating the release of hormones such as human growth hormone and insulin-like growth factor 1 [37] and supporting muscle growth. Furthermore, BFR training encourages the recruitment of fast-twitch muscle fibers, which are essential for gains in muscle strength and hypertrophy [38].

Although studies utilizing 20% 1RM BFR training (with bilateral occlusion) for 8 weeks did not elicit a significant overall increase in muscle mass, there was a significant increase in muscle mass observed in the left leg. This finding aligns with the hypothesis put forward by prior studies [5], which suggests that localized pressure training promotes muscle mass growth in the pressurized region but may not lead to noticeable changes in overall muscle mass due to the relatively small proportion of localized muscle mass. Notably, the left-leg muscle mass growth exceeded that of the right-leg across all groups, potentially due to initial differences in muscle mass and strength, neuromuscular adaptations, and psychological factors.

For adults with limited training experience, the non-dominant leg (typically the left leg) may demonstrate greater growth potential under identical training stimuli, starting from a lower baseline. Additionally, weaker muscles undergoing novel training stimuli undergo more significant neuromuscular adaptations, and trainees may focus more intently on and exert greater effort towards training these weaker muscle groups, thereby facilitating their growth.

### 4.3. Comparative Analysis of Changes in Lower-Limb Thigh Circumference

In this study, we selected the thigh circumference measured at its midpoint as the indicator for thigh girth, a commonly utilized parameter that reflects the size of the thigh area [39]. By contrasting the BFR and HL-RT groups, which employed distinct pressure application techniques, we found that thigh circumference increased post-training, albeit mostly not to a statistically significant degree. Specifically, within the HL-RT group, there was a statistically significant increase in left-leg thigh circumference, whereas the increase in right-leg thigh circumference did not attain statistical significance. Current research findings on the impacts of BFR training and HL-RT on thigh circumference are mixed. For instance, Luebbers et al. [40] noted increased thigh circumference in both BFR and HL-RT groups after a 7-week intervention, yet neither increase reached statistical significance. Conversely, another study reported significant increases in thigh circumference in the BFR (30% 1RM) group, as well as an increase in right-thigh circumference in the HL-RT (70% 1RM) group, following a 4-week resistance training intervention [5]. These discrepancies may stem from variations in training intensity, participant characteristics, and the duration of training. In our study, notable changes in lower-limb circumference were predominantly observed in the left leg, potentially due to the exercise load design. The relatively low exercise load used here (20% 1RM versus 70% 1RM) may have elicited a more prominent stimulatory effect on the left leg compared to other studies’ intensities. Additionally, participants’ gender could be a contributing factor, as females generally exhibit smaller changes in muscle morphology.

In this study, we compared the effects of BFR and HL-RT, with different pressure application methods, on limb circumference. HL-RT, a well-established and effective exercise modality, has been shown to enhance muscle function and augment skeletal muscle volume [18,41]. Limb circumference, a crucial indicator of muscle volume, is closely tied to changes in muscle mass. Our experimental results indicated that the left-leg thigh circumference change rates in the I-BFR, A-BFR, and HL-RT groups were 0.7%, 2.1%, and 4.4%, respectively, while the corresponding rates for the right leg were 0.3%, 0.7%, and 0.8%. The greater increase in left-thigh circumference in the BFR group compared to the right is consistent with previous studies conducted among young female athletes, where the BFR group’s left-thigh circumference increased more than the right [42]. Considering the trends in muscle mass changes, the variations in limb thigh circumference observed in this study are largely dependent on muscle mass fluctuations, thereby supporting the strong correlation between limb thigh circumference and muscle volume.

When comparing the two BFR training groups that utilized different pressure application methods, the A-BFR group exhibited higher percentage changes in both left- and right-leg thigh circumference compared to the I-BFR group. This suggests that applying a higher cuff pressure stimulus may facilitate increases in limb thigh circumference. Notably, even in the I-BFR group, where occlusion pressure was incrementally increased, the gains in thigh circumference were inferior to those achieved in the A-BFR group. Thus, an initial high-intensity stimulus appears to play a crucial role in promoting muscle growth and increasing thigh circumference, which may not be fully compensated for by gradually increasing pressure thereafter. However, it is worth noting that the sample size in this study is relatively small, and this finding is speculative based on experimental data. Therefore, further research is necessary to validate the underlying mechanisms. Future studies could delve into the specific effects of different pressure strategies on muscle growth and thigh circumference increases, as well as how these effects vary across different populations.

### 4.4. Limitation

Despite striving for rigor in its design and implementation, this study has some limitations. Firstly, the study applied the same cuff pressure (160–220 mmHg) to all subjects, a practice that, while conventional in the fields of exercise training and general fitness, inevitably introduces individual differences. These differences may result in varying benefits for certain subjects during the training process, thereby affecting the universality and accuracy of the study results.

Secondly, this study did not utilize advanced equipment such as Doppler ultrasound for blood flow measurement, instead relying on blood pressure and heart rate monitoring to ensure the safety of subjects. While this method is effective in most cases, it may not precisely capture subtle variations in blood flow, thus limiting our deeper understanding of the training effects.

## 5. Conclusions

While A-BFR and I-BFR did not display statistically noteworthy differences in overall training outcomes, A-BFR exhibited greater potential. Following an 8-week intervention period, both A-BFR and I-BFR delivered muscle strength enhancements and body composition improvements that were on a par with HL-RT. However, HL-RT demonstrated more significant improvements in leg circumference.

## Figures and Tables

**Table 1:** Volunteer’s basic condition.

Group	N (F/M)	Weight (Kg)	Height (cm)
I-BFR	8/3	60.51 ± 10.90	167.27 ±7.43
A-BFR	8/3	54.67 ± 6.00	167.18 ± 6.06
HL-RT	8/3	59.09 ± 8.86	166.09 ± 8.58

Values are mean ± SD. I-BFR, incremental blood flow restriction; A-BFR, absolute blood flow restriction; HL-RT, high-load resistance training; F, Female; M, male.

**Table 2:** Change in Maximal Strength of lower limb muscle groups after training intervention.

	Group	Pre-Intervention	Post-Intervention	*p*	Cohen’s d
LP-1RM/Kg	I-BFR	143.05 ± 49.42	158.75 ± 50.45 **	<0.001	2.987
	A-BFR	127.10 ± 51.45	142.26 ± 51.87 **	<0.001	2.516
	HL-RT	125.03 ± 37.29	140.24 ± 37.16 **	<0.001	2.198
KF-1RM/Kg	I-BFR	56.36 ± 26.49	63.20 ± 27.24 **	<0.001	1.522
	A-BFR	49.04 ± 17.05	57.53 ± 18.90 **	<0.001	2.413
	HL-RT	50.37 ± 18.46	59.09 ± 23.84 **	<0.001	2.147
KE-1RM/Kg	I-BFR	95.97 ± 35.73	106.42 ± 36.92 **	<0.001	2.225
	A-BFR	90.99 ± 24.79	104.78 ± 26.28 **	<0.001	1.594
	HL-RT	99.06 ± 22.90	111.79 ± 25.40 **	0.003	1.192

Values are mean ± SD. I-BFR, incremental blood flow restriction; A-BFR, absolute blood flow restriction; HL-RT, high-load resistance training; LP, leg press; KF, knee flexion; KE, knee extension; ** *p* ≤ 0.01 vs. pre.

**Table 3:** Changes in FFM, BMI and Muscle Mass after training intervention.

	Group	Pre-Intervention	Post-Intervention	*p*	Cohen’s d
FFM/Kg	I-BFR	46.97 ± 10.88	46.11 ± 10.01	0.273	0.350
	A-BFR	42.88 ± 5.80	43.35 ± 6.65	0.492	0.215
	HL-RT	45.82 ± 8.85	45.84 ± 7.95	0.975	0.010
BMI	I-BFR	21.78 ± 2.10	22.05 ± 1.82	0.096	0.553
	A-BFR	19.54 ± 1.74	19.94 ± 1.89	0.135	0.491
	HL-RT	21.06 ± 2.18	21.07 ± 2.44	0.923	0.030
MMT/kg	I-BFR	43.51 ± 9.60	44.52 ± 10.23	0.140	0.483
	A-BFR	40.37 ± 5.58	40.90 ± 6.38	0.400	0.265
	HL-RT	43.01 ± 8.65	43.17 ± 7.73	0.831	0.066
MML/Kg	I-BFR	7.73 ± 2.17	8.30 ± 1.94 **	0.008	0.989
	A-BFR	7.19 ± 1.03	7.73 ± 1.18 **	0.007	1.013
	HL-RT	7.61 ± 1.14	8.29 ± 1.72 *	0.027	0.784
MMR/Kg	I-BFR	8.24 ± 2.33	8.29 ± 2.06	0.487	0.217
	A-BFR	7.57 ± 0.94	7.81 ± 1.18	0.055	0.653
	HL-RT	8.06 ± 1.51	8.30 ± 1.70	0.170	0.445

Values are mean ± SD. I-BFR, incremental blood flow restriction; A-BFR, absolute blood flow restriction; HL-RT, high-load resistance training; FFM, fat-free mass; BMI, body mass index; MMT, total muscle mass; MML, left leg muscle mass; MMR, right leg muscle mass; ** *p* ≤ 0.01 vs. pre; * *p* ≤ 0.05 vs. pre.

**Table 4:** Changes in Bone Mineral Density and Bone Mineral Content after training intervention.

	Group	Pre-Intervention	Post-Intervention	*p*	Cohen’s d
BMD-L/g/cm^2^	I-BFR	0.44 ± 0.07	0.48 ± 0.12	0.283	0.342
	A-BFR	0.45 ± 0.09	0.46 ± 0.08	0.132	0.495
	HL-RT	0.47 ± 0.08	0.47 ± 0.09	0.579	0.173
BMC-L/g/cm^2^	I-BFR	0.42 ± 0.07	0.46 ± 0.11	0.316	0.319
	A-BFR	0.41 ± 0.10	0.42 ± 0.1	0.088	0.570
	HL-RT	0.44 ± 0.07	0.45 ± 0.08	0.584	0.171
BMD-R/g/cm^2^	I-BFR	0.47 ± 0.076	0.49 ± 0.128	0.634	0.148
	A-BFR	0.46 ± 0.11	0.47 ± 0.10	0.475	0.224
	HL-RT	0.46 ± 0.08	0.46 ± 0.11	0.958	0.016
BMC-R/g/cm^2^	I-BFR	0.44 ± 0.07	0.46 ± 0.12	0.607	0.160
	A-BFR	0.40 ± 0.14	0.43 ± 0.11	0.187	0.427
	HL-RT	0.44 ± 0.13	0.42 ± 0.15	0.620	0.154

Values are mean ± SD. I-BFR, incremental blood flow restriction; A-BFR, absolute blood flow restriction; HL-RT, high-load resistance training; BMD-L, bone mineral density of left root bone; BMC-L, bone mineral content of left root bone; BMD-R, bone mineral density of right root bone; BMC-L, bone mineral content of right root bone.

**Table 5:** Changes in Lower Limb Thigh circumference after training intervention.

	Group	Pre-Intervention	Post-Intervention	*p*	Cohen’s d
TCL/cm	I-BFR	50.34 ± 5.64	50.71 ± 4.41	0.642	0.144
	A-BFR	48.21 ± 3.7	49.25 ± 3.45	0.166	0.450
	HL-RT	47.43 ± 4.19	49.50 ± 4.77 **	0.008	1.003
TCR/cm	I-BFR	50.66 ± 5.49	50.86 ± 4.33	0.810	0.074
	A-BFR	48.90 ± 3.64	49.28 ± 3.71	0.658	0.138
	HL-RT	48.15 ± 4.46	48.81 ± 4.97	0.307	0.324

Values are mean ± SD. I-BFR, incremental blood flow restriction; A-BFR, absolute blood flow restriction; HL-RT, high-load resistance training; TCL, Left thigh circumference; TCR, right thigh circumference ** *p* ≤ 0.01 vs. pre.

## Data Availability

The raw data supporting the conclusions of this article are available from the first author upon reasonable request.

## References

[1] Libardi C.A., Chacon-Mikahil M.P., Cavaglieri C.R., Tricoli V., Roschel H., Vechin F.C., Conceição M.S., Ugrinowitsch C. (2015). Effect of concurrent training with blood flow restriction in the elderly. Int. J. Sports Med..

[2] Centner C., Jerger S., Lauber B., Seynnes O., Friedrich T., Lolli D., Gollhofer A., König D. (2022). Low-load blood flow restriction and high-load resistance training induce comparable changes in patellar tendon properties. Med. Sci. Sports Exerc..

[3] Yasuda T., Ogasawara R., Sakamaki M., Ozaki H., Sato Y., Abe T. (2011). Combined effects of low-intensity blood flow restriction training and high-intensity resistance training on muscle strength and size. Eur. J. Appl. Physiol..

[4] Yu L., Zhao Z., Wang Z., Gao J., Zhu X. (2020). Effects of short-term kaatsu training on body composition and cardiovascular function in male adults. J. Beijing Sport Univ..

[5] Li Z., Zhao Z., Wang M., Chen C., Wei W., Liang Y. (2019). Effect of 4 Weeks KAATSU Training on Body Composition and Maximum Strength of Male Handball Players. China Sport Sci. Technol..

[6] Su R. (2019). Study on the practical application and physiological mechanism of blood flow restriction training in promoting muscle fitness. Chin. J. Rehabil. Med..

[7] Brandner C.R., Clarkson M.J., Kidgell D.J., Warmington S.A. (2019). Muscular Adaptations to Whole Body Blood Flow Restriction Training and Detraining. Front. Physiol..

[8] Martín-Hernández J., Marín P.J., Menéndez H., Ferrero C., Loenneke J.P., Herrero A.J. (2013). Muscular adaptations after two different volumes of blood flow-restricted training. Scand. J. Med. Sci. Sports.

[9] Wei J., Li B., Yang W., Wang X., Feng L., Li Y. (2019). The effects and mechanisms of blood flow restriction training. China Sport Sci..

[10] Loenneke J.P., Fahs C.A., Wilson J.M., Bemben M.G. (2011). Blood flow restriction: The metabolite/volume threshold theory. Med. Hypotheses.

[11] Nakajima T., Kurano M., Iida H., Takano H., Oonuma H., Morita T., Meguro K., Sato Y., Nagata T., KAATSU Training Group (2006). Use and safety of KAATSU training:Results of a national survey. Int. J. KAATSU Train. Res..

[12] Vanwye W.R., Weatherholt A.M., Mikesky A.E. (2017). Blood Flow Restriction Training: Implementation into Clinical Practice. Int. J. Exerc. Sci..

[13] Martín-Hernández J., Santos-Lozano A., Foster C., Lucia A. (2018). Syncope Episodes and Blood Flow Restriction Training. Clin. J. Sport Med..

[14] Takada S., Okita K., Suga T., Omokawa M., Kadoguchi T., Sato T., Takahashi M., Yokota T., Hirabayashi K., Morita N. (2012). Low-intensity exercise can increase muscle mass and strength proportionally to enhanced metabolic stress under ischemic conditions. J. Appl. Physiol..

[15] Patterson S.D., Hughes L., Warmington S., Burr J., Scott B.R., Owens J., Abe T., Nielsen J.L., Libardi C.A., Laurentino G. (2019). Blood flow restriction exercise: Considerations of methodology, application, and safety. Front. Physiol..

[16] Che T., Yang T., Liang Y., Li Z. (2021). Effects of low-intensity semi-squat kaatsu training on activation degree of core muscle groups and rating of perceived exertion. China Sport Sci..

[17] Early K.S., Rockhill M., Bryan A., Tyo B., Buuck D., McGinty J. (2020). Effect of blood flow restriction training on muscular performance, pain and vascular function. Int. J. Sports Phys. Ther..

[18] Horiuchi M., Stoner L., Poles J. (2023). The effect of four weeks blood flow restricted resistance training on macro- and micro-vascular function in healthy, young men. Eur. J. Appl. Physiol..

[19] Bemben D.A., Sherk V.D., Buchanan S.R., Kim S., Sherk K., Bemben M.G. (2022). Acute and chronic bone marker and endocrine responses to resistance exercise with and without blood flow restriction in young men. Front. Physiol..

[20] Thiebaud R.S., Loenneke J.P., Fahs C.A., Rossow L.M., Kim D., Abe T., Anderson M.A., Young K.C., Bemben D.A., Bemben M.G. (2013). The effects of elastic band resistance training combined with blood flow restriction on strength, total bone-free lean body mass and muscle thickness in postmenopausal women. Clin. Physiol. Funct. Imaging.

[21] Chang H., Yan J., Lu G., Chen B., Zhang J. (2023). Muscle strength adaptation between high-load resistance training versus low-load blood flow restriction training with different cuff pressure characteristics: A systematic review and meta-analysis. Front. Physiol..

[22] Ozaki H., Yasuda T., Ogasawara R., Sakamaki-Sunaga M., Naito H., Abe T. (2013). Effects of high-intensity and blood flow-restricted low-intensity resistance training on carotid arterial compliance: Role of blood pressure during training sessions. Eur. J. Appl. Physiol..

[23] Brzycki M. (1993). Strength testing—Predicting a one-rep max from reps-to-fatigue. J. Phys. Educ. Recreat. Dance.

[24] Centner C., Jerger S., Lauber B., Seynnes O., Friedrich T., Lolli D., Gollhofer A., König D. (2023). Similar patterns of tendon regional hypertrophy after low-load blood flow restriction and high-load resistance training. Scand. J. Med. Sci. Sports.

[25] Karabulut M., Abe T., Sato Y., Bemben M.G. (2010). The effects of low-intensity resistance training with vascular restriction on leg muscle strength in older men. Eur. J. Appl. Physiol..

[26] Lixandrão M.E., Ugrinowitsch C., Laurentino G., Libardi C.A., Aihara A.Y., Cardoso F.N., Tricoli V., Roschel H. (2015). Effects of exercise intensity and occlusion pressure after 12 weeks of resistance training with blood-flow restriction. Eur. J. Appl. Physiol..

[27] Lixandrão M.E., Ugrinowitsch C., Berton R., Vechin F.C., Conceição M.S., Damas F., Libardi C.A., Roschel H. (2018). Magnitude of muscle strength and mass adaptations between high-load resistance training versus low-load resistance training associated with blood-flow restriction: A systematic review and meta-analysis. Sports Med..

[28] Loenneke J.P., Kim D., Fahs C.A., Thiebaud R.S., Abe T., Larson R.D., Bemben D.A., Bemben M.G. (2015). Effects of exercise with and without different degrees of blood flow restriction on torque and muscle activation. Muscle Nerve.

[29] Yasuda T., Brechue W.F., Fujita T., Shirakawa J., Sato Y., Abe T. (2009). Muscle activation during low-intensity muscle contractions with restricted blood flow. J. Sports Sci..

[30] Letieri R.V., Teixeira A.M., Furtado G.E., Lamboglia C.G., Rees J.L., Gomes B.B. (2018). Effect of 16 weeks of resistance exercise and detraining comparing two methods of blood flow restriction in muscle strength of healthy older women: A randomized controlled trial. Exp. Gerontol..

[31] Yasuda T., Abe T., Brechue W.F., Iida H., Takano H., Meguro K., Kurano M., Fujita S., Nakajima T. (2010). Venous blood gas and metabolite response to low-intensity muscle contractions with external limb compression. Metabolism.

[32] Teixeira E.L., Barroso R., Silva-Batista C., Laurentino G.C., Loenneke J.P., Roschel H., Ugrinowitsch C., Tricoli V. (2018). Blood flow restriction increases metabolic stress but decreases muscle activation during high-load resistance exercise. Muscle Nerve.

[33] Yasuda T., Fukumura K., Fukuda T., Iida H., Imuta H., Sato Y., Yamasoba T., Nakajima T. (2014). Effects of low-intensity, elastic band resistance exercise combined with blood flow restriction on muscle activation. Scand. J. Med. Sci. Sports.

[34] Wernbom M., Paulsen G., Bjørnsen T., Cumming K., Raastad T. (2021). Risk of Muscle Damage With Blood Flow-Restricted Exercise Should Not Be Overlooked. Clin. J. Sport Med..

[35] Tomohiro Y., Miwa M., Yoshiaki S., Toshiaki N. (2017). Use and safety of KAATSU training: Results of a national survey in 2016. Int. J. KAATSU Train. Res..

[36] Garber C.E., Blissmer B., Deschenes M.R., Franklin B.A., Lamonte M.J., Lee I.M., Nieman D.C., Swain D.P. (2011). American college of sports medicine position stand. quantity and quality of exercise for developing and maintaining cardiorespiratory, musculoskeletal, and neuromotor fitness in apparently healthy adults: Guidance for prescribing exercise. Med. Sci. Sports Exerc..

[37] Hwang P.S., Willoughby D.S. (2019). Mechanisms behind blood flow-restricted training and its effect toward muscle growth. J. Strength Cond. Res..

[38] Pearson S.J., Hussain S.R. (2015). A review on the mechanisms of blood-flow restriction resistance training-induced muscle hypertrophy. Sports Med..

[39] Niu Y., Qiao Y., Fan Y. (2020). Effects of blood flow restriction training on muscle morphology and function of subjects: A meta-analysis. J. Cap. Univ. Phys. Educ. Sports.

[40] Luebbers P.E., Fry A.C., Kriley L.M., Butler M.S. (2014). The effects of a 7-week practical blood flow restriction program on well-trained collegiate athletes. J. Strength Cond. Res..

[41] Damas F., Libardi C.A., Ugrinowitsch C., Vechin F.C., Lixandrão M.E., Snijders T., Nederveen J.P., Bacurau A.V., Brum P., Tricoli V. (2018). Early- and later-phases satellite cell responses and myonuclear content with resistance training in young men. PLoS ONE.

[42] Che T. (2021). Effect of Lower Limb Kaatsu Training Combined with High Intensity Resistance Training on the Muscle Strength of Core Area and Lower Limb of Adolescent Female Wrestlers. Master’s Thesis.

